# High resolution structural evidence suggests the Sarcoplasmic Reticulum forms microdomains with Acidic Stores (lysosomes) in the heart

**DOI:** 10.1038/srep40620

**Published:** 2017-01-17

**Authors:** Daniel Aston, Rebecca A. Capel, Kerrie L. Ford, Helen C. Christian, Gary R. Mirams, Eva A. Rog-Zielinska, Peter Kohl, Antony Galione, Rebecca A. B. Burton, Derek A. Terrar

**Affiliations:** 1 Department of Pharmacology, University of Oxford, Mansfield Road, Oxford, OX1 3QT, UK; BHF Centre of Research Excellence, University of Oxford, UK; 2 Department of Physiology, Anatomy and Genetics, Sherrington Building, University of Oxford, Sherrington Road, Oxford, OX1 3PT, UK; BHF Centre of Research Excellence, University of Oxford, UK; 3 Computational Biology, Department of Computer Science, Wolfson Building, University of Oxford, Oxford, OX1 3QD, UK; 4 National Heart and Lung Institute, Imperial College London, London, UK; 5 Institute for Experimental Cardiovascular Medicine, University Heart Centre Freiburg/Bad Krozingen, University Medical School, Freiburg, Germany

## Abstract

Nicotinic Acid Adenine Dinucleotide Phosphate (NAADP) stimulates calcium release from acidic stores such as lysosomes and is a highly potent calcium-mobilising second messenger. NAADP plays an important role in calcium signalling in the heart under basal conditions and following β-adrenergic stress. Nevertheless, the spatial interaction of acidic stores with other parts of the calcium signalling apparatus in cardiac myocytes is unknown. We present evidence that lysosomes are intimately associated with the sarcoplasmic reticulum (SR) in ventricular myocytes; a median separation of 20 nm in 2D electron microscopy and 3.3 nm in 3D electron tomography indicates a genuine signalling microdomain between these organelles. Fourier analysis of immunolabelled lysosomes suggests a sarcomeric pattern (dominant wavelength 1.80 μm). Furthermore, we show that lysosomes form close associations with mitochondria (median separation 6.2 nm in 3D studies) which may provide a basis for the recently-discovered role of NAADP in reperfusion-induced cell death. The trigger hypothesis for NAADP action proposes that calcium release from acidic stores subsequently acts to enhance calcium release from the SR. This work provides structural evidence in cardiac myocytes to indicate the formation of microdomains between acidic and SR calcium stores, supporting emerging interpretations of NAADP physiology and pharmacology in heart.

The traditional view that lysosomes function solely as a terminal end of cellular catabolic pathways has been challenged recently by various lines of evidence showing that this tiny acidic organelle also plays a central role in cell function via lysosomal calcium signalling^1^. Lysosomal calcium signalling is a little-studied pathway in the heart which has been implicated in the adrenergic responses of both atrial^2^ and ventricular^3^ myocytes. Nicotinic Acid Adenine Dinucleotide Phosphate (NAADP) is a second messenger which induces the release of calcium from acidic stores such as the lysosome and lysosome-related reserve granules in the sea urchin egg^4^. The importance that this signalling molecule plays in the mobilisation of calcium in many species of animal and plant has been established in recent decades^5^. NAADP-mediated calcium release has been shown to be involved in a remarkably broad range of cellular signalling processes including fertilisation^6^, insulin secretion and responses^7^,^8^ and neuronal differentiation^9^. Further, recent work has implicated the pathway in infectivity of the Ebola virus^10^.

With every heartbeat, excitation-contraction coupling converts the passage of electrical excitation across the myocardium to contraction via a rapid rise and subsequent recovery of cytosolic calcium, the so-called ‘calcium transient’. Calcium entering the cytosol via voltage-gated L-type calcium channels is sensed at the cytosolic face of ryanodine receptors (RyR), which then open, leading to massive calcium release from the sarcoplasmic reticulum (SR). This amplification from the cell’s intracellular calcium store is known as calcium induced calcium release (CICR) and is terminated by closure of ryanodine receptors and clearance of calcium from the cytosol back into the SR. This clearance occurs through active pumping into the SR by the sarco-endoplasmic reticulum calcium ATPase (SERCA) and passive exchange out of the cell through the sodium calcium exchanger (NCX). SERCA activity is modulated by phospholamban (PLB) which binds to and inhibits SERCA under resting conditions. Phosphorylation of PLB relieves this inhibition, thus accelerating clearance of calcium from the cytosol and increasing SR calcium content.

Several calcium channels also reside in the lysosomal membrane and studies have explored the role of these lysosomal calcium channels in both physiological and pathological cellular processes^11^. Action of NAADP at Two-Pore Channel-2 (TPC2) channels in the lysosomal membrane is the most likely mechanism by which NAADP-mediated calcium release occurs, this having been demonstrated in both isolated membrane and organelle studies and a number of tissue types^10^,^12^,^13^,^14^,^15^,^16^,^17^,^18^,^19^. Indeed, NAADP no longer produces a response in cardiac myocytes after abolition of the H^+^ gradient required for lysosomal calcium loading^20^, and cells derived from TPC2 knock-out mice do not respond to NAADP application^3^. Antibody-mediated blocking studies have been used to propose Transient Receptor Potential Mucolipin 1 (TRPML1) as the calcium-permeable channel responsible for NAADP-mediated calcium release^21^. In SKBR3 cells these channels coimmunoprecipitate, however overexpression of TRPML1 had no effect on NAADP responses whereas TPC2 overexpression was associated with an enhancement of NAADP-mediated calcium signals^18^. Further, pancreatic acinar cells lacking the TRPML1 protein exhibit no difference in NAADP-evoked calcium oscillations^18^. Alternatively, it has been suggested that NAADP may act directly at the RyR to cause calcium release^22^,^23^.

Recent work has demonstrated that the NAADP signalling pathway has a vital physiological role in the heart^2^,^3^,^20^,^24^. Release of NAADP into atrial or ventricular cardiomyocytes results in an enhancement of the calcium transient, positive inotropic (enhanced contraction) and lusitropic (enhanced relaxation) effects and increased calcium loading of the SR^20^. Conversely, acute inhibition of the pathway using bafilomycin-A1 to disrupt acidic store function or Ned-19, a specific NAADP receptor antagonist^25^, causes a reduction in the calcium transient. The use of bafilomycin-A1, Ned-19, or genetic knockout of the TPC2 protein (*Tpcn2*^*−/−*^) results in significant blunting of responses to isoprenaline in terms of calcium transient and inotropic effect, suggesting that NAADP/TPC2 plays an important role in the transduction of β-adrenergic stimulation^3^. Furthermore, *Tpcn2*^*−/−*^ mice are partially protected from the cardiac arrhythmias and ventricular hypertrophy associated chronic β-adrenergic stress^3^, and either knock-out of TPC2^3^ or inhibition of the NAADP pathway^24^ significantly reduces spontaneous activity associated with acute β-adrenergic stress.

Although it is possible that calcium release from acidic stores and from the SR are simply additive, several lines of evidence suggest that an amplification mechanism is present. Enhanced calcium loading of the SR has been observed following NAADP release^2^,^20^, and the increase in calcium transient amplitude that occurs due to NAADP can be blocked by inhibitors of type-II calmodulin-dependent protein kinase (CaMKII) – suggesting that this serine/threonine protein kinase is required for regulation of the calcium transient amplitude by NAADP^3^. Calcium liberated from the lysosome could also directly promote CICR from RyR, or result in enhanced SR calcium release indirectly, perhaps via phosphorylation of the ryanodine receptor (RyR) by CaMKII. If changes in RyR activity were the sole mechanism, elevated calcium loading of the SR would not be expected. It is possible, however, that effects of NAADP in the heart involve both calcium re-uptake and calcium release mechanisms simultaneously.

It has previously been shown that the effects of NAADP-mediated calcium release are specific to the SR and do not affect L-type-calcium currents on the surface membrane^2^. Our previous evidence suggests that calcium released from the lysosome in response to NAADP action at TPC2 alters SERCA and/or RyR via modulation^2^,^20^ of CaMKII activity^3^. An obvious prerequisite to this hypothesis is a subcellular spatial organisation which brings the membranes of acidic organelles such as endosomes and lysosomes into close enough apposition with the SR to allow calcium release from acidic stores to raise local concentrations enough to modulate CaMKII, SERCA, PLB and/or RyR activity of the SR.

The existence of cellular regions between two membranes that are in close proximity (typically less than 30 nm separation) favours congregation of calcium signalling proteins in such restricted spaces, and these regions are functionally referred to as microdomains or nanojunctions^26^,^27^. These regions have sometimes been referred to as membrane contact sites (MCS)^27^. These MCS allow the segregation of sometimes incompatible biochemical reactions into specific compartments with tailored microenvironments and form the anatomical basis for functional calcium signalling^27^,^28^,^29^,^30^. Such microdomains between lysosomes and the ER have recently been described in pulmonary artery smooth muscle^31^,^32^,^33^ and human fibroblasts^34^. Here, we look at the intimate associations of lysosomes in the heart in the context of what is already known about NAADP signalling in this tissue. We examined the location and organisation of these acidic organelles with respect to calcium signalling membranes, such as the SR and T-tubules using both 2D electron microscopy and 3D electron tomographic reconstructions. This information is important for constraining functional models of the calcium signalling roles of NAADP in the heart at rest and it is relevant to the contribution of NAADP signalling in the β-adrenergic response. Further, we investigated the proximity of lysosomes to mitochondria, in light of recent evidence supporting the role of NAADP signalling via TPC1 in ischaemia/reperfusion injury^35^.

## Results

### Electron Microscopy of Lysosomes Identifies Potential Nanojunctions for Calcium Signalling in Cardiac Ventricular Myocytes

**Figure 1:**
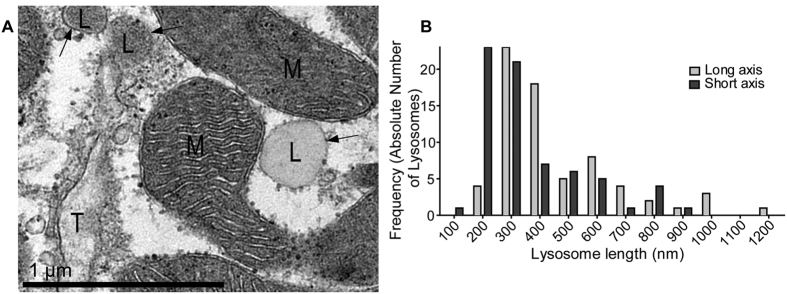
Identification of lysosomes in cardiac ventricular myocytes. (**A**) Electron micrograph of rabbit ventricular tissue containing both ‘electron dense’ and ‘electron lucent’ lysosomes (L). Both forms were used for analysis described in text and subsequent figures. Note that both are darker than the surrounding cytoplasm, and have a clearly defined single lipid bilayer membrane (indicated by black arrows). Mitochondria (M) and a T-tubule (T) are also marked. (**B**) Dimensions of lysosomes analysed in this study.

In order to investigate the proximity of lysosomal membranes to other subcellular organelles, we carried out transmission electron microscopy (TEM) on rabbit isolated ventricular myocytes and fixed ventricular tissue. A total of 69 lysosomes were identified in TEM images of rabbit ventricular cells. Although their appearance was heterogeneous, they could be identified by characteristic morphological features^36^. The lysosomal luminal matrix is bounded by a bilayer membrane, and we observed matrices with varying electron densities similar to the light and dark lysosomes previously described. Intra-lysosomal matrices were sometimes uniform and only slightly more electron dense than the cytoplasm, and in other cases there was a significant difference in density and uniformity (see example Fig. 1A for a micrograph containing both types). This could be related to the age of the animals, which determines the number of dense bodies and lipofuscin inclusions^26^ as well as some contributions from experimental methods (such as fixation methods or post staining of sections)^37^.

Measured lysosomes (n = 69) were found to have a median long axis of 383 nm (306–580 nm) and a median short axis of 290 nm (220–455 nm). This is in broad agreement with recently reported lysosomal dimensions in pulmonary artery smooth muscle cells^32^. It has been previously noted that differentiation between endosomes and small lysosomes is difficult and that a threshold diameter of 200 nm should be used to define a lysosome^32^. Application of this criterion to the data presented here results in a total of 61 lysosomes imaged with a median long axis of 414 nm (325–598 nm) and short axis of 320 nm (248–483 nm). A summary of these data is presented in Fig. 1B. All further analyses were performed without application of this criterion. Other structures observed in our TEM analysis included multivesicular bodies, lipid droplets and autophagosomes, which were not included in our analysis (see Supplementary Figure 1 for examples).

**Figure 2:**
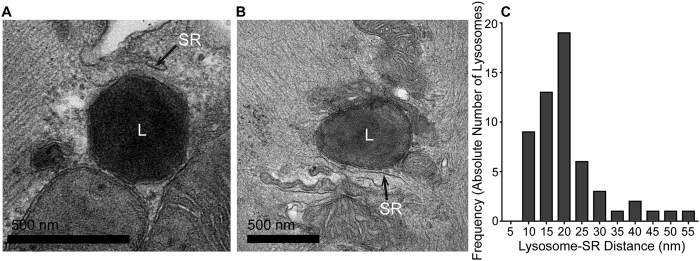
Lysosomes are in close proximity to the sarcoplasmic reticulum (SR) in rabbit ventricular myocytes, forming nanojunctions. (**A**,**B**) Representative examples of lysosomes (L) in close proximity to the SR. (**C**) Population data showing the shortest distance from a lysosomal-related organelle membrane to the nearest SR membrane. Median distance 20 nm (13–24 nm), n = 56.

Two dimensional transmission electron microscopy (TEM) is likely to provide an over-estimation of the distance between two structures, due to the fact that the plane and angle of the section is unlikely to always include the shortest distance between them. Nevertheless, TEM images of isolated rabbit ventricular myocytes demonstrated that the majority of lysosomes (56/69) were closely associated with SR membrane (see examples Fig. 2A and B). The median distance between these 56 organelles and the closest piece of visible SR was 20 nm (13–24 nm). Of these, 48 were close enough (<30 nm separation) to define as an MCS^38^. Population data are presented in Fig. 2C.

**Figure 3:**
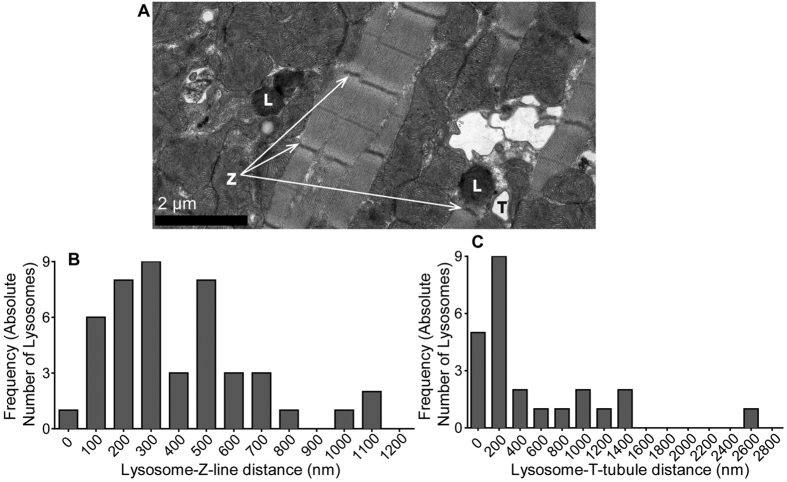
Measured distances from lysosomes to T-tubules and Z-lines. (**A**) Representative wide-field TEM images illustrating lysosomes (L) in relation to T-tubules (T) and Z-lines (Z). (**B**) Population data illustrating the distance between a lysosomes and the nearest Z-line. Average distance 318 nm (176–529 nm), n = 45. (**C**) Population data illustrating the distance between a lysosome and the nearest T-tubule. Average distance 163 nm (123–870 nm), n = 24.

Next we measured the shortest apparent distances between lysosomes and the nearest T-tubule and Z-line, where these were also visible on the micrographs used above. A representative example including both T-tubules and Z-lines can be seen in Fig. 3A. For both these structures, the nearest distance was much further away than the nearest SR membrane; 318 nm (176–529 nm) for Z-lines (n = 45) and 163 nm (123–870 nm) for T-tubules (n = 24; One-way ANOVA with Sidak’s correction, P < 0.0001 for L-SR distance vs L-TT distance and also P < 0.0001 for L-SR distance vs L-Z distance). The distributions were positively skewed but not binomial in nature (Fig. 3B and C respectively). In 23 cases, lysosomes were seen with adjacent SR, and a T-tubule could also be seen in the same micrograph. In all cases, the distance to the SR was less than the distance to the T-tubule.

**Figure 4:**
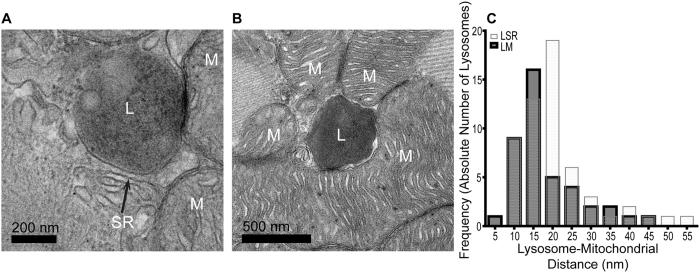
Lysosomes are in close proximity to mitochondria in cardiac ventricular myocytes, feasibly forming nanojunctions. (**A**,**B**) Representative examples of lysosome (L) in contact with (**A**) and in close proximity to (**B**) mitochondria (M) in isolated rabbit ventricular cardiomyocytes. SR = Sarcoplasmic Reticulum, arrow indicates nearest membrane to L. (**C**) Population data showing the shortest distances from a lysosomal-related organelle membrane to the nearest mitochondrial membrane (dark grey bars). Average distance 17 nm (14–37 nm), n = 51. These data include only instances in which a distinct gap was measurable between the two organelles in order to exclude confounding from mitochondria entering the degradative pathway. The L-SR distance histogram is included for comparison (light bars).

As well as being in close proximity with SR membrane, there were many examples where lysosomes were seen in close contact with mitochondria (n = 51). Of these 51 cases, 42 were also associated with SR and are therefore included in the analyses above. The distances from a lysosome to the nearest mitochondrion varied considerably; some were so close that it was not possible to resolve a gap between the lysosomal membrane and the mitochondrial membrane (Fig. 4A). As it is possible that lysosomes touching mitochondria could be involved in autophagy, we have noted their existence but did not include them in numerical analyses. Although the maximum measured distance between a lysosome and the nearest mitochondrial membrane was 1419 nm, the majority of organelles in this data set were closely associated with mitochondria (representative examples presented in Fig. 4B) and only five were more than 75 nm away from their nearest mitochondrial neighbour. The minimum measurable distance was 7.1 nm. After removal of statistical outliers (ROUT, Prism 6), the median lysosome-mitochondrial distance was 17 nm (14–37 nm, n = 41). Summary data are presented as a frequency histogram together with L-SR analysis for comparison (Fig. 4C).

Conventional TEM methods utilising higher concentrations of glutaraldehyde and followed by Spurr resin embedding allow for better visualisation of membranes. Whilst this method is ideal for imaging intracellular membranes, it does not allow for immuno-gold labelling of proteins as the glutaraldehyde form hydroxyl-methylene bridges between reactive end-groups of adjacent protein chains, which can diminish immunoreactivity or accessibility to immunoreactive sites. For this reason, we used sections from LR White embedded tissue (tissue fixation with 4% PFA and 0.1% glutaraldehyde) to perform immuno-gold labelling of the lysosome-specific protein LAMP-2. Supplementary Figure 2 shows immuno-gold labelling of LAMP-2, as a lysosomal marker, in LR White embedded mouse ventricular tissue. Gold-labelled organelles are seen in close proximity to probable SR membrane and mitochondria, consistent with our numerical analyses above.

### Three-Dimensional Electron Tomography of Lysosomes Confirms Extensive Structures Suitable to Form Signalling Microdomains

2D imaging techniques suffer from systematic over-estimation of proximity measurements. We therefore carried out 3D electron tomography imaging and analysis in order to gather an accurate picture of the spatial interrelation of organelles within the cell and to identify the true minimum distances between lysosomal membranes and other subcellular calcium signalling structures.

**Figure 5:**
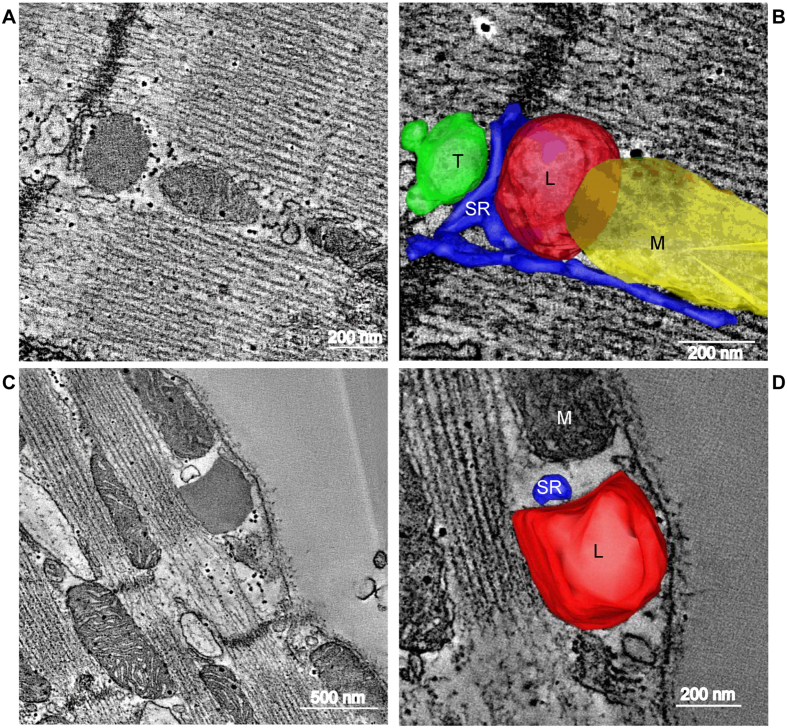
3D Electron Tomography reconstruction of lysosomes, SR, T-tubules and mitochondria. Representative electron tomography (ET) images of lysosomes near calcium signalling organelles, including raw images (**A**,**C**) and reconstructed organelles in 3D (**B**,**D**) of sarcoplasmic reticulum (**A** to **D**), mitochondria and T-tub (**A**,**B**) in rabbit left ventricular tissue. Dual-axis ET and IMOD software were used to image, reconstruct and model lysosomes (L, red), sarcoplasmic reticulum (SR, blue), mitochondria (M, yellow), and T-tubules (T, green) in 3D. Isovolumetric voxel size = 1.206 nm, Z-depth = 275 nm. Scale bars (**A**,**B**,**D**) = 200 nm and (C) = 500 nm. See Supplementary Video 1 for 3D animation of tomographic reconstruction of cell in (**A**,**B**).

Figure 5 shows representative examples of individual sectioning planes (A&C), and 3D reconstructions of lysosomes and surrounding organelles (B&D). After removal of outliers, consistent with the analysis method of our 2D TEM study, the median distance (gap width) between lysosomes and the SR was 3.3 nm (see Table 1 for detail on minimum distances to mitochondria and T-tubules). A video reconstruction together with multi-angle views of the cell in Fig. 5A and B is available in the supplementary information (Supplementary Video 1).

### Confocal Microscopy in Fixed Cells Identifies Sarcomeric Periodicity to LAMP-2 Protein Expression in Cardiac Ventricular Myocytes

**Figure 6:**
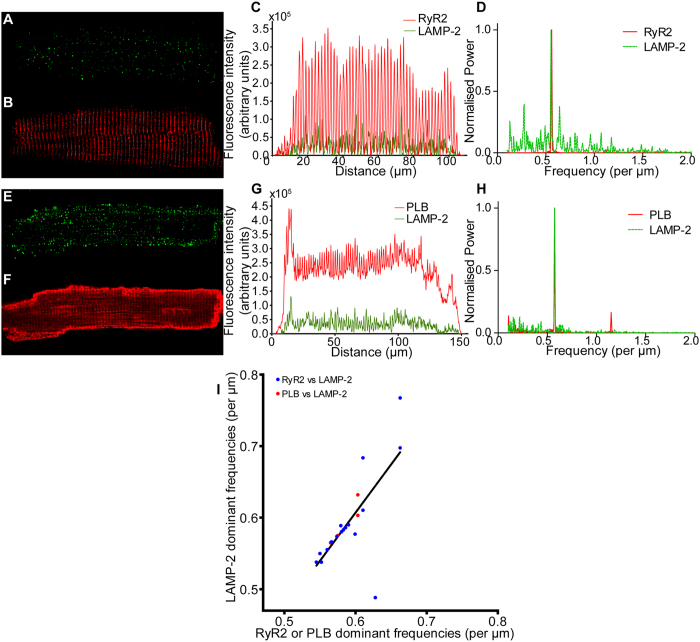
Lysosomes are localised in a periodic pattern with sarcomeric distribution, similar to RyR and PLB, in fixed, isolated rabbit ventricular myocytes. (Top line) Representative example of LAMP-2 and RyR2 co-labelling experiment. Immunolabelling of LAMP-2 (**A**) and RyR2 (**B**) co-stained in the same, fixed, myocyte. (**C**) Intensity plot illustrating total pixel intensity for each column of pixels, for the two stains. Results of Fourier analyses (**D**) indicate a dominant frequency of 0.56 striations μm^−1^, or a signal peak every 1.79 μm, for RyR2 and LAMP-2. (Middle line) Representative example of LAMP-2 and PLB co-labelling experiment. Confocal images of LAMP-2 (**E**) and PLB (**F**) with merged column intensity plot of both stains (**G**). Fourier analysis (**H**) of each signal indicate a dominant frequency of 0.57 signal peaks μm^−1^ for both PLB and LAMP-2, equivalent to a peak every 1.74 μm. (Bottom Line) (**I**) A correlation plot of dominant frequencies for LAMP-2 against either RyR2 or PLB in co-labelling experiments reveals strong correlation (Pearson product-moment correlation co-efficient (r) = 0.72, n = 22 cells (n = 18 for RyR2 vs LAMP-2 (blue) and n = 4 for PLB vs LAMP-2 (pink)).

We next utilised immunocytochemical staining of isolated rabbit ventricular myocytes to investigate whether acidic calcium stores exhibit a regular spatial organisation at the micro-scale within whole ventricular myocytes. Several anti-TPC2 antibodies were tested, and all showed non-specific staining in TPC2 knock-out mice (data not shown). We concluded that, at present, no commercially-available antibody is sufficiently robust for immuno-localisation of the TPC2 channel. We therefore stained for LAMP-2, a lysosomal membrane protein with roles in organelle stability, chaperone-mediated autophagy and lysosomal motility. LAMP-2 staining revealed a punctate distribution (Fig. 6A and E). Staining against type-2 ryanodine receptor (RyR2) or phospholamban (PLB) revealed the expected regular appearance (for example Fig. 6B and F respectively)^39^: both of these labels were seen as striations running across the width of the cell, perpendicular to its long axis. To determine any non-specific labelling of secondary antibodies control cells with no primary antibody, incubated with anti-rabbit or anti-mouse secondaries alone were imaged and found to give no detectable signal (data not shown).

The punctate staining of lysosomes in immunocytochemistry (eg Fig. 6A and D) has not previously been studied to assess the possibility that they are arranged in non-random patterns. Fourier analysis of fluorescence intensity measurements, summed perpendicularly to the cells’ long axis, was used to determine the dominant frequency of the signal corresponding to each protein in order to determine whether lysosomes occur with any periodicity and how this relates to other proteins of the SR. An example of a RyR2 and LAMP-2 labelled cell and another of a PLB and LAMP-2 labelled cell with analyses for these images are given in Fig. 6. Confocal images were first rotated so that striations ran vertically (Fig. 6B and F) and total fluorescence was calculated for each vertical column of pixels (Fig. 6C and G), such that in a 100 × 100 pixel image the intensities of all 100 pixels positioned vertically at the horizontal position of x = 1 are added together to give a fluorescence intensity in arbitrary units, and similar summations are made for all horizontal positions up to x = 100; these fluorescence values are plotted against horizontal distance along the cell perpendicular to the direction of striations. Fourier analysis was then carried out on the resulting intensity vs distance waveforms to ascertain the dominant frequency of each signal (Fig. 6D and H). Fourier analysis of a total of 22 cells (18 RyR2 vs LAMP-2 and 4 PLB vs LAMP-2) revealed a strong positive correlation between the dominant frequencies for RyR2 and PLB when plotted against LAMP-2 (r = 0.72, Fig. 6I). In the RyR2-labelled cell displayed, dominant frequencies for both RyR2 and LAMP-2 were 0.56 signal peaks per μm, or one every 1.80 μm (Fig. 6A to D). The PLB-labelled cell exhibited dominant frequencies for both PLB and LAMP-2 of 0.57 signal peaks per μm, or one every 1.74 μm (Fig. 6E to H). Data from a total of 18 cells dual-stained for LAMP-2 and RyR2 yielded an average (mean ± SEM) of 0.592 ± 0.015 and 0.590 ± 0.008 signal peaks per μm for each protein respectively, corresponding to a peak-to-peak distance of 1.69 μm. Data from a total of 4 cells dual-stained for LAMP-2 and PLB yielded an average of 0.594 ± 0.015 and 0.587 ± 0.010 signal peaks per μm respectively, corresponding to a peak-to-peak distance of 1.68 and 1.70 μm for LAMP-2 and PLB respectively in this data set. All of these values are within the expected range for a sarcomere of an isolated rabbit ventricular cardiomyocyte^40^.

**Figure 7:**
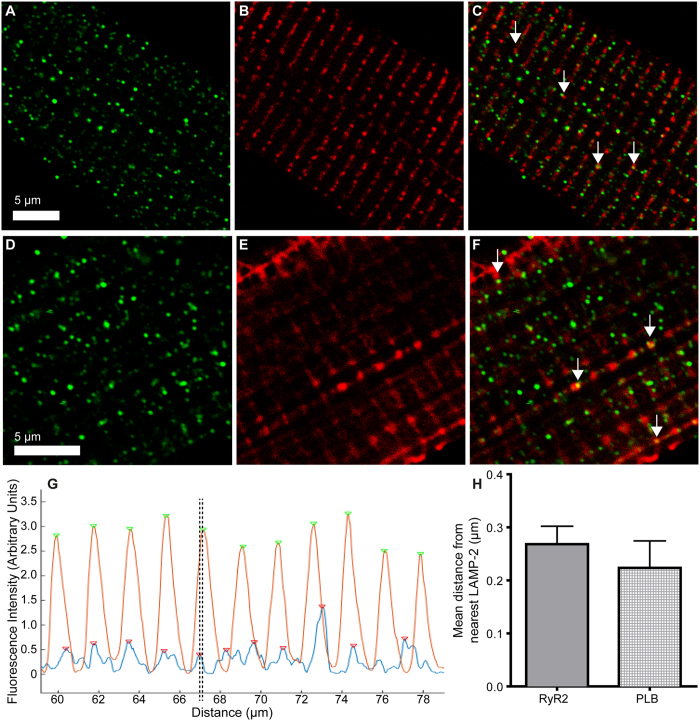
LAMP-2 and RyR2 or PLB are closely aligned but not co-localised in fixed, isolated rabbit ventricular myocytes. (Top line) Representative immunolabelling of LAMP-2 (**A**) and RyR2 (**B**). (**C**) Merged image. White arrows indicate areas of close proximity. (Middle line) Representative immunolabelling of LAMP-2 (**D**) and PLB (**E**). (**F**) Merged image. White arrows indicate areas of close proximity. (Bottom line) Calculation of distance between fluorescence peaks of LAMP-2 and either RyR2 or PLB in immunolabelling experiments. (**G**) Column fluorescence intensity data for a representative cell stained for LAMP-2 and RyR2 after processing with the peak-finding algorithm. Green arrows indicate peaks of RyR2, red arrows of LAMP-2 (detected by automated MatLab script). Dotted lines indicate an example of a pair of RyR2 and LAMP-2 peaks for distance measurements. (**H)** Population data to show average distance in μm between LAMP-2 and either RyR2 (0.27 ± 0.03 μm, n = 16) or PLB (0.22 ± 0.05 μm, n = 3).

Despite this striking similarity between the signal frequencies of LAMP-2 and RyR2 or PLB, there was minimal spatial co-localisation between them. Pearson correlation coefficients for each pixel intensity for LAMP-2 vs RyR2 (representative cell Fig. 7A to C) and LAMP-2 vs PLB (representative cell Fig. 7D to F) were 0.42 ± 0.03 and 0.22 ± 0.06 respectively, indicating little overlap in the fluorescence. However, in both groups of cells there were areas where the signals from the two proteins came into very close proximity (for example Fig. 7C and F, white arrows). The mean distance between LAMP-2 and RyR2 or PLB was estimated by measuring the offset between the fluorescence intensity peaks of the two signals using the column intensity data described above. This gave a mean separation of 0.27 ± 0.03 μm between LAMP-2 and RyR2 (n = 16 cells) and 0.22 ± 0.05 μm between LAMP-2 and PLB (n = 3 cells). See Fig. 7G and H.

### Confocal Microscopy: in Live Cells Confirms NAADP Binding Sites on Acidic Organelles in Cardiac Ventricular Myocytes

Ned-19 is a synthetic NAADP receptor-specific antagonist^25^ which is also tryptophan-derived and therefore fluorescent. Indeed, Ned-19 has been shown to inhibit binding of NAADP to its receptor, preventing the release of calcium in response to NAADP^41^ and it can be used to localise the position of NAADP receptors in live cells^42^.

**Figure 8:**
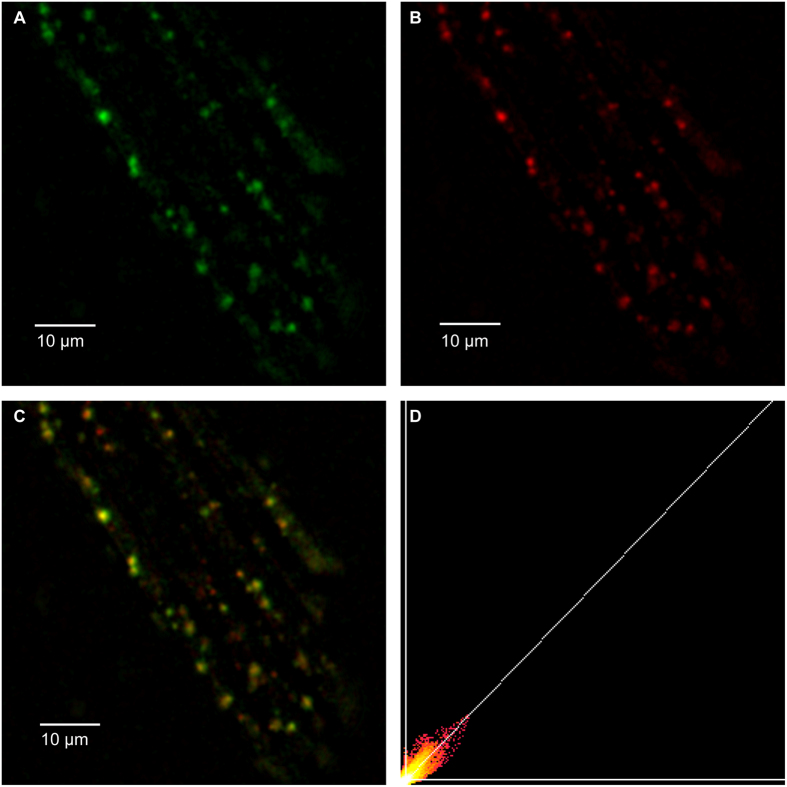
NAADP binding sites co-label with acidic calcium stores in live rabbit ventricular myocytes. (**A**) Punctate staining of Ned-19 (100 μM, 40 min), a fluorescent NAADP antagonist which binds at the NAADP receptor. (**B**) LysoTracker red (500 nM, 2 min) labelling of acidic organelles. (**C**) Merged image of (**A**,**B**) shows a high degree of co-localisation between Ned-19 and acidic stores. Images taken of live cells in Tyrode solution at 37 °C. (**D**) Correlation plot of A and B fluorescence for each pixel. Pearson correlation coefficient of 0.91 for this cell. PMCC for all cells was 0.69 ± 0.04 (n = 8). See Supplementary Figures 3 and 4 for control experiments.

Live isolated rabbit ventricular cardiomyocytes were incubated with Ned-19 (100 μM, Fig. 8A) and LysoTracker red (500 nM, Fig. 8B), a commercially-available stain which is fluorescent when inside acidic compartments. There was a high degree of co-localisation between the signals (Fig. 8C and D, Pearson’s r = 0.69 ± 0.04, n = 8), confirming probable localisation of NAADP receptors on acidic organelles such as lysosomes, and the potential for NAADP-mediated calcium release from these organelles in live cells. Control experiments confirmed that LysoTracker signals did not bleed through into the Ned-19 channel and that cellular autofluorescence could not account for punctate signals under UV excitation (see Supplementary Figure 3). The incubation of cells with BZ-194, another (non-fluorescent) antagonist of NAADP prior to the addition of Ned-19 abolished the fluorescent signal, suggesting a specific binding site for these compounds, rather than accumulation as part of a degradative pathway (see Supplementary Figure 4).

## Discussion

Previous work has presented strong functional evidence for a role of NAADP as a calcium-mobilising second messenger in cardiac myocytes^2^,^3^,^20^. The results described in this paper provide clear structural evidence for nanojunctions between lysosomes and both the SR and mitochondria in rabbit ventricular myocytes; observations of the ultrastructural organisation which support the plausibility of earlier functional hypotheses^2^,^3^,^20^,^35^,^43^. Further, we have used Ned-19 and LysoTracker staining in live cells to localise NAADP-binding sites and showed that NAADP binding is restricted to acidic organelles in cardiac myocytes.

Transmission electron microscopy (TEM) demonstrated that L-SR microdomains exist in the heart. Lysosomes in our TEM data have dimensions similar to those from previous publications^32^ and exhibit the expected range of electron densities^36^. These methods have great utility in measuring membrane distances between organelles, however there are limitations to be considered in such techniques. We accept that some organelles included in our analysis may include late endosomes and potentially other lysosome-related organelles, however, re-assuringly immuno-gold labelling of the lysosome-specific protein LAMP-2 identified organelles in similar close proximity to the SR and mitochondria. Our findings of a median L-SR distance in the ventricle of 20 nm certainly falls within the expected range required for a functional microdomain identified by Fameli *et al*.’s modelling^32^,^44^,^45^. As 2D images tend to overestimate minimum distances, further 3D tomography work was carried out to identify more accurate spatial interrelations between the organelles. Ten lysosomes were imaged in our 3D study, the median minimum distance to the nearest piece of SR was a mere 3.3 nm (for comparison – the distance between t-tubules and SR is ~20 nm).

A probable contribution of NAADP-mediated acidic store calcium signalling to whole-cell calcium transients in the heart has been established for nearly ten years, since the first paper published^20^. This signalling is completely abolished in mice lacking TPC2 channels^3^ and appears to require both acidic stores and an intact SR^2^,^20^, suggesting that calcium released from lysosomes affects SR calcium content and release. A role for lysosome-derived calcium released via TPC2 has also been implicated in other cell types, including the stimulation of calcium release from the ER in pancreatic beta cells^13^, granule exocytosis in T-cells^46^ and protein trafficking in the endolysosomal degradation pathway of hepatocytes^47^.

We show localisation of the NAADP receptors on acidic stores in cardiac ventricular myocytes using a live cell microscopy method. Ned-19 is a fluorescent functional antagonist of NAADP discovered using virtual screening for molecules with structures and charge densities similar to that of NAADP itself^25^. It has been shown to prevent binding of NAADP to its endogenous binding site and prevents the release of calcium in response to NAADP application^41^. There was a high degree of co-localisation between Ned-19 and LysoTracker red labelling in cardiomyocytes, suggesting that NAADP binds to acidic organelles in these cells. The pattern of Ned-19 staining, punctate and distributed throughout the cell, was similar to that published in rat astrocyte studies^42^. Some areas adjacent to the nucleus were stained with LysoTracker red but did not co-label with Ned-19. It is possible that they represent Golgi apparatus or that some of the range of acidic organelles^48^ may not take part in NAADP signalling. Localisation of NAADP binding to acidic stores is in keeping with our^2^,^3^,^20^ and other^35^,^49^ previous studies in the heart. Staining controls show that Ned-19 images did not arise from signal bleed-through. The natural course of cell uptake and/or degradation for some compounds is itself through the endo-lysosomal pathway. Indeed, it is thought that the Ebola virus utilises this process in order to infect cells^10^. In the presence of BZ194, an NAADP antagonist based on nicotinic acid, Ned-19 signals were no longer seen on acidic organelles. This suggests that Ned-19 is binding to a specific site as opposed to accumulating within acidic organelles as part of the degradative pathway. The molecular identity of the NAADP receptor still remains a controversial issue as acknowledged by Dammermann *et al*.^50^. It is interesting to note that BZ194 has been proposed to antagonise an NAADP-mediated increase in RyR1 opening directly at RyRs in Jurkat T lymphocytes^50^. A previous study^51^ has shown that modification of NAADP at the nicotinic acid moiety results in compounds which bind the sea urchin NAADP receptor with altered agonist potency and can directly antagonise the binding of NAADP itself. Published studies^24^,^50^,^52^,^53^,^54^ have thus far not investigated the binding of BZ194 at other proposed NAADP binding sites however its actions, both previously published and in this paper, are consistent with effects at NAADP receptors regardless of their subcellular site.

TEM measurements of the distance between a lysosome and the nearest T-tubule reveal an average of 163 nm, which is in reasonable agreement with the distance measured between RyR2 and LAMP-2 using immunofluorescence (268 nm). However, this is too large to suggest a signalling microdomain between these organelles in cardiac ventricular myocytes, and this is not entirely unexpected, as the functional consequences of lysosomal-related calcium release appear to be restricted to the SR; photorelease of NAADP has no effect on L-type calcium current^2^,^20^ and the effect of isoprenaline on L-type calcium currents is not different in the absence of NAADP-mediated signalling^3^. Both RyR2 and phospholamban are proteins found on the SR membrane. RyR in cardiac ventricular myocytes are situated on the SR membrane, forming couplons at sites of T-tubule contact^55^. The assumption that antibody labelling of LAMP-2 will always result in a point-spread function centred on the lysosomal membrane nearest to the T-tubule or SR cannot be made in these experiments and an average LAMP-2 to RyR or PLB distance as measured by immunofluorescence would be expected to fall at a distance between the closest and farthest membranes, taking into account staining on lysosomal membrane that is immediately adjacent to the SR as well as that on the far side.

Subjecting LAMP-2 fluorescence signals to Fourier analysis of fluorescence intensities, summed perpendicularly to the length of the cell, reveals a defined periodicity to acidic organelle positioning in cardiac cells which is consistent with a sarcomeric pattern; the LAMP-2 dominant frequency of 0.59 signals per μm compares favourably with the expected sarcomeric spacing in these cells^40^. The periodicity of LAMP-2 staining observed during our experiments is very similar to that observed for both RyR2 and PLB, and in some cells it was identical. In order to further investigate this relationship, phase lags were artificially introduced to one of the signals, and the Pearson correlation coefficients were recalculated. The effect of this is shown in Supplementary Figure 5, which illustrates that the correlation is at a maximum when the introduced lag is equal to the mean distance between LAMP-2 and RyR2 or PLB. We therefore conclude that the signal peaks are well defined but separated by a small amount, equal to the mean distance between the LAMP-2 and either RyR2 or PLB proteins, and this pattern repeats every sarcomere.

In many cell types, acidic store calcium signalling leads directly to calcium release from the endoplasmic reticulum^38^. An all-or-nothing, ‘quantal’ calcium release pattern has been postulated to account for lysosomal calcium release in these cell types leading, if a threshold of quanta are released, to calcium-induced calcium release and calcium oscillations^56^. Cells conforming to this ‘trigger’ hypothesis can have a trigger zone, in which acidic calcium stores are tightly packed in an appropriate signalling domain, for instance near RyR3 in pulmonary artery smooth muscle cells^57^. Our immunolabelling data suggests that, in rabbit cardiac myocytes, lysosomes are also far from random in their positioning, exhibiting periodicity consistent with the length of a sarcomere. Our data do not suggest that all RyR or PLB are associated with lysosomes, but that a subset of the area of the SR forms a functional calcium signalling microdomain with lysosomes. Unlike in smooth muscle, this subset does not form a defined, single ‘trigger zone’ but is distributed throughout the cell.

Given the lysosome-SR distances measured by our TEM study, it is perhaps surprising that neither RyR nor PLB were seen to frequently co-localise with lysosomal membrane protein LAMP-2 in our immunocytochemistry study. Indeed, with the resolution limit of standard confocal microscopy, proteins on membranes that are a median distance of 3.3 nm apart would be expected to be indistinguishable. It is reasonable to assume that labelling of LAMP-2 will not always be seen on the membrane closest to the SR, as mentioned above, and it may be that the number of ‘co-localised’ areas is artificially lowered due either to clustering of signalling proteins at the SR nanojunction^30^, i.e. presence of TPCs at the expense of LAMP-2, or difficulty in multiple antibodies with a size of ~10 nm^58^ penetrating the junctional spaces which we have measured to be < 10 nm at their nearest point. It is not yet known whether all lysosomes contribute to NAADP signalling, however a lack of clear co-localisation in immunocytochemistry could indicate that only a subset of lysosomes is involved. In our TEM measurements, 48 of 69 lysosomes analysed were found in proximity with SR which could be defined as a membrane contact site, however cutting in a 2D plane cannot rule out that SR was in close apposition in other areas of the organelle. In 3D tomography measurements, all lysosomes imaged formed these MCS relationships with surrounding SR.

It was recently shown that NAADP acting via TPC1 channels may contribute to ischaemia-reperfusion injury in cardiac ventricular myocytes^35^. Interactions between lysosomes and mitochondria have been observed in other tissues^59^, and in cardiac atria^60^. Analysis of our TEM images showed several instances where the distance between a lysosome and a mitochondrion was so small that a gap could not be resolved (which could be possible consequence of the section thickness (80 nm) and the angle of sectioning through the membrane). Autophagic processes may explain some of these interactions; it is well known that mitochondria are recycled by the endolysosomal system at the end of their useful life^61^. We did not include instances of lysosomes where membranes were deforming around, or were continuous with, those of adjacent mitochondria. While it is certainly possible that some of these close interactions represent lysosomes about to begin autophagy, the average measured distance of 17 nm in TEM data between mitochondrial membrane and lysosome membrane is highly supportive of microdomain signalling between these organelles. The nature of the images generated by 3D tomography suggests that even this small distance may be an over-estimate, as with L-SR separation, identifying a median distance of 6.2 nm between a lysosome and the closest mitochondrion.

Hearts from mice lacking TPC1, or exposed to NAADP receptor antagonists Ned-19 or Ned-K, have been shown to be partially protected from ischaemia-reperfusion injury. This was hypothesised to arise by prevention of calcium oscillations that cause opening of the mitochondrial permeability transition pore (mPTP)^35^. Opening of mPTP is known to be related to apoptotic and necrotic cell death^62^, such as during reperfusion after ischaemic injury in the heart^63^. Nanojunctions found between ER and mitochondria are subjected to calcium concentrations much higher than those of the total cytosol during exposure to appropriate stimuli^64^. Spatial proximity between the ER and mitochondria appears to underlie the rapid rise of mitochondrial calcium seen in response to IP_3_R opening in rat liver cells, being abolished if tethering structures are artificially lengthened^65^. It is also of note that when mitochondria-ER tethers were engineered to be < 5 nm, mitochondrial calcium uptake after ER IP_3_R opening also led to mitochondrial calcium overload and mPTP opening^65^. It seems plausible, therefore, that close association between lysosomes and mitochondria could be of functional significance with relation to the same pathway – an aspect that will have to be explored in future research.

In summary, this paper represents the first description of nanojunctions between acidic calcium stores and the SR in mammalian cardiomyocytes. This is in keeping with previous work on the same signalling system in pulmonary artery smooth muscle^32^,^33^ and human fibroblasts^34^. Together with confirmation of co-localisation between NAADP binding sites and acidic stores, this provides a structural context for hypotheses, generated from pharmacological studies in cardiac ventricular tissue, that suggest a role for NAADP in augmenting CICR upon β-adrenergic stimulation^20^,^43^ by increasing SR calcium concentration without modulating L-type calcium channel currents^2^,^20^.

## Methods

All animal experiments were performed in accordance with the United Kingdom Home Office Guide on the Operation of Animal (Scientific Procedures) Act of 1986. All experimental protocols (Schedule 1) were approved by the University of Oxford.

### Rabbit ventricular cell isolation

Cardiomyocytes were isolated using a method modified from Powell, *et al*.^66^.

Briefly, Male New Zealand White rabbits (approximately 1 kg) were sacrificed by intravenous overdose of sodium pentobarbitone. The heart was rapidly excised, washed in heparin-containing solution (5 U/mL), and mounted on a Langendorff apparatus for perfusion with zero-calcium solution containing (in mM) NaCl 136, KCl 5.4, NaHCO_3_ 12, sodium pyruvate 1, NaH_2_PO_4_ 1, MgCl_2_ 1, EGTA 0.04, glucose 5, gassed with 95% O_2_/5% CO_2_ to maintain pH 7.4. After 2 min, digestion was initiated using an identical solution containing 0.5 mg.ml^−1^ type II collagenase (Worthington Biochemical Corp, Lakewood, NJ, USA) and 100 μM CaCl_2_ instead of EGTA. This was allowed to recirculate for 30–35 minutes after which cells were mechanically isolated, filtered, centrifuged and suspended in Dulbecco’s Modified Eagle Medium (DMEM), or fixed within 60 min for electron microscopy and immunocytochemistry.

### Electron microscopy

Samples (from n = 3 animals) were prepared from either chemically fixed isolated myocytes or from chemically fixed intact ventricular tissue.

Rabbit ventricular cardiomyocytes isolated using the method described above, or approximately 1 mm^3^ pieces of tissue from ventricles were rapidly dissected (post Langendorff perfusion) and fixed in Karnovsky fixative (paraformaldehyde 4%, glutaraldehyde 5%, cacodylate buffer 80 mM, pH 7.4, Karnovsky)^67^ and were embedded in Spurr’s resin^68^. Sections were cut to approximately 80 nm thickness (Reichart Ultracut) and post-stained to achieve contrast by use of 2% aqueous uranyl acetate and Reynolds lead citrate. Images were obtained using a transmission electron microscope (TEM, Joel 1200EX II). Analysis and measurements were made using Gatan DigitalMicrograph and ImageJ software.

Lysosomes were identified by their previously well-documented appearance; that is, a matrix slightly more electron dense than the surrounding cytoplasm enclosed by a single lipid bilayer membrane^32^, see Fig. 1. Measurements from both cells and tissue were included in analysis and the subcellular appearance of these two preparations was indistinguishable under electron microscopy.

### Electron tomography (ET)

Rabbit left ventricular tissue fragments (n = 6 animals), were fixed via Langendorff perfusion in iso-osmotic (300 mOsm) Karnovsky’s reagent (2.4% sodium cacodylate, 0.75% paraformaldehyde, 0.75% glutaraldehyde), were washed with 0.1 M sodium cacodylate, post-fixed in 1% OsO_4_ for 1 h, dehydrated in graded acetone, and embedded in Epon-Araldite resin. Thick sections were cut (~275 nm, 3 sections per animal), transferred onto copper slot grids coated with 1% formvar and post-stained with 2% aqueous uranyl acetate, followed by Reynolds’ lead citrate. Colloidal gold particles (15 nm) were added to both surfaces of the sections to serve as fiducial markers for subsequent tilt series alignment. All sections were prepared and imaged at the Boulder Laboratory for 3D Electron Microscopy of Cells (University of Colorado, Boulder, CO), using an intermediate voltage electron microscope (Tecnai TF30; 4FEI-Company, Eindhoven, The Netherlands) operating at 300 kV, with images captured on a charge-coupled device camera (UltraScan; Gatan, Pleasanton, CA), at a pixel size of (1.206 nm^2^).

For dual-axis tomography, series of tilted views were collected from + 60° to −60° at 1° increments at 20,000x magnification. After the first tilt series was acquired, the specimen was rotated by 90° in the horizontal plane, and another + 60° to −60° tilt series was taken. Images from each tilt-series were aligned by fiducial marker tracking and back-projected to generate two single full-thickness reconstructed volumes (tomograms), which were then combined to generate a high-resolution 3D reconstruction of the original volume of interest. Tomograms were processed and analysed using the IMOD software, also used to segment and generate 3D models of the structures of interest^69^,^70^. The models were smoothed and meshed to obtain a final 3D representation, in which spatial relationships of T-tubules, sarcoplasmic reticulum, mitochondria with lysosomes can be visualised.

### Immunocytochemistry

Isolated rabbit ventricular cardiomyocytes were fixed within 60 minutes of isolation. Briefly, cells were allowed to settle for 20 minutes on flamed glass coverslips. The cells were fixed with 4% paraformaldehyde solution (Sigma, UK), before permeablisation with 0.2% Triton-X 100 (Sigma-Aldrich, Dorset, UK) and blocked with 5% donkey serum. Cells were labelled with primary antibodies overnight at 4 °C (rabbit-anti-LAMP-2, Thermofisher PA1-655, Lot: PA189864, 1:100; mouse-anti-RyR2, Abcam ab2827, 1:100; mouse-anti-phospholamban, Abcam ab2865, 1:100). Secondary antibodies were diluted in 5% donkey serum (donkey-anti-rabbit-AlexaFluor-488, Thermofisher A-21206; donkey-anti-mouse-Dylight-555, Newmarket Scientific AS12-1935), and incubated at room temperature for 2 h (control cells where the primary or secondary was to be excluded were incubated with 5% donkey serum alone without addition of the relevant antibody). Coverslips were mounted using Vectashield Hardset with DAPI (Vectorlabs, Peterborough, UK) and sealed.

Slides were stored in the dark at 4 °C, and imaged within 48 h using an Olympus Fluoview FV1200 confocal microscope. Images obtained using the Olympus FV1200 confocal microscope with Fluoview software were not deconvolved and Kalman averaging of four pictures was used to obtain each final image. A 60x oil-immersion low-chromatic aberration objective was used and the confocal aperture was set to one Airy unit.

### Analysis of images obtained using immunocytochemistry

Confocal images were analysed using custom MatLab (Mathworks, Cambridge, UK) scripts written in-house. Briefly, cell orientation was automatically detected and rotated to vertically align striations of RyR2 or PLB before thresholding to remove background noise. Total intensity for each vertical pixel column was calculated and waveforms subjected to a Fourier transform using the MatLab Signal Processing Toolbox ‘periodogram’ command to determine component frequencies. To analyse the distance between adjacent staining, signal waveforms were overlaid and the pixel-wise distance between the maxima of each pair of peaks was calculated. DAPI fluorescence signals were subjected to the same process as a control; no sarcomeric signal was detectable for this stain. MatLab code has been published on Figshare under the doi:10.6084/m9.figshare.3102769.

### Live cell confocal microscopy

Isolated rabbit ventricular myocytes were incubated with 100 μM Ned-19 for 40 minutes at room temperature with addition of 500 nM LysoTracker red for the final 2 min. Cells were allowed to adhere to a glass coverslip and superfused with Tyrode’s solution containing (in mM): 135 NaCl, 4.5 KCl, 11 glucose, 20 HEPES, 1 MgCl2, 1.8 CaCl2, pH 7.4 with NaOH. Ned-19 fluorescence was excited at 355 nm and detected at 415 ± 30 nm (experimental approach similar to that described in Barcelo-Torns *et al*.^42^), whilst LysoTracker red was excited at 514 nm and collected at 590–747 nm.

Imaging was performed with a Leica SP5 inverted confocal microscope with a 40x or 63x oil-immersion objective. To analyse co-localisation, images were smoothed with a Gaussian filter (radius 2 pixels) and median image intensity (36 × 36 pixel) subtracted from the smoothed image to remove background noise. Pearson’s correlation coefficients were calculated from resulting images using ImageJ coloc2 plugin.

### Statistics

Data is presented as mean ± standard error for normally distributed data and median (interquartile range) for skewed or non-parametric groups. Outliers were identified using ROUT analysis with Q = 1% (GraphPad Prism 6). Pearson’s product-moment correlation coefficient (r) was calculated to compute the degree of correlation between two variables.

## Additional Information

**How to cite this article:** Aston, D. *et al*. High resolution structural evidence suggests the Sarcoplasmic Reticulum forms microdomains with Acidic Stores (lysosomes) in the heart. *Sci. Rep.* **7**, 40620; doi: 10.1038/srep40620 (2017).

**Publisher's note:** Springer Nature remains neutral with regard to jurisdictional claims in published maps and institutional affiliations.

## Supplementary Material

Supplementary Information

Supplementary Video 1

## Figures and Tables

**Table 1:** 3D tomography-based analysis of spatial interactions of lysosomes and other organelle membrane systems in rabbit left ventricular cells (n = 6 animals).

Lysosome	Diameter min in [nm]	Diameter max in [nm]	Min dist to SR in [nm]	Min dist to Z line in [nm]	Min dist to M line in [nm]	Min dist to T-tubule in [nm]	Min dist to mitochondria in [nm]
1	166.1	202.3	3.3	232.0	462.4	16.3	3.2
2	143.9	258.9	4.1	435.3	108.1	441.9	262.7§
3	146.0	233.1	1.1	145.6	524.8	145.1	28.4
4	187.0	315.5	5.4	n/a	236.9	n/a	1.7
5	467.5	528.2	5.4	455.0	332.1	n/a	69.1
6	291.1	386.1	2.2	76.8	652.3	52.4	5.1
7	204.6	380.4	15.6§	376.7	221.3	8.8	17.8
8	327.6	358.6	1.1	354.1	320.4	n/a	7.2
9	340.6	420.2	2.3	39.1	606.7	213.3	4.5
10	441.2	442.3	3.6	51.5	682.2	n/a	n/a
Median	247.9	369.5	3.3	232.0	397.3	98.8	6.2

Measurements were performed using IMOD software^69^. n/a – no accurate measurement possible, § indicates statistical outlier (ROUT analysis, Q = 1%, GraphPad Prism 6). Medians are given after removal of statistical outliers, in keeping with the analysis of 2D TEM measurements.

## References

[b1] Medina D. L. . Lysosomal calcium signalling regulates autophagy through calcineurin and TFEB. Nature cell biology 17, 288–299, doi: 10.1038/ncb3114 (2015). 25720963 PMC4801004

[b2] Collins T. P., Bayliss R., Churchill G. C., Galione A. & Terrar D. A. NAADP influences excitation-contraction coupling by releasing calcium from lysosomes in atrial myocytes. Cell calcium 50, 449–458, doi: 10.1016/j.ceca.2011.07.007 (2011). 21906808

[b3] Capel R. A. . Two-pore Channels (TPC2s) and Nicotinic Acid Adenine Dinucleotide Phosphate (NAADP) at Lysosomal-Sarcoplasmic Reticular Junctions Contribute to Acute and Chronic beta-Adrenoceptor Signaling in the Heart. The Journal of biological chemistry 290, 30087–30098, doi: 10.1074/jbc.M115.684076 (2015). 26438825 PMC4705968

[b4] Churchill G. C. . NAADP mobilizes Ca(2+) from reserve granules, lysosome-related organelles, in sea urchin eggs. Cell 111, 703–708 (2002). 12464181 10.1016/s0092-8674(02)01082-6

[b5] Hooper R. & Patel S. NAADP on target. Adv Exp Med Biol 740, 325–347, doi: 10.1007/978-94-007-2888-2_14 (2012). 22453949

[b6] Churchill G. C. . Sperm deliver a new second messenger: NAADP. Current biology: CB 13, 125–128 (2003). 12546785 10.1016/s0960-9822(03)00002-2

[b7] Johnson J. D. & Misler S. Nicotinic acid-adenine dinucleotide phosphate-sensitive calcium stores initiate insulin signaling in human beta cells. Proceedings of the National Academy of Sciences of the United States of America 99, 14566–14571, doi: 10.1073/pnas.222099799 (2002). 12381785 PMC137923

[b8] Masgrau R., Churchill G. C., Morgan A. J., Ashcroft S. J. & Galione A. NAADP: a new second messenger for glucose-induced Ca2+ responses in clonal pancreatic beta cells. Current biology: CB 13, 247–251 (2003). 12573222 10.1016/s0960-9822(03)00041-1

[b9] Brailoiu E. . Messenger-specific role for nicotinic acid adenine dinucleotide phosphate in neuronal differentiation. The Journal of biological chemistry 281, 15923–15928, doi: 10.1074/jbc.M602249200 (2006). 16595650

[b10] Sakurai Y. . Ebola virus. Two-pore channels control Ebola virus host cell entry and are drug targets for disease treatment. Science (New York, N.Y.) 347, 995–998, doi: 10.1126/science.1258758 (2015). PMC4550587 25722412

[b11] Morgan A. J., Platt F. M., Lloyd-Evans E. & Galione A. Molecular mechanisms of endolysosomal Ca2+ signalling in health and disease. The Biochemical journal 439, 349–374, doi: 10.1042/bj20110949 (2011). 21992097

[b12] Zong X. . The two-pore channel TPCN2 mediates NAADP-dependent Ca(2+)-release from lysosomal stores. Pflugers Archiv: European journal of physiology 458, 891–899, doi: 10.1007/s00424-009-0690-y (2009). 19557428 PMC2719734

[b13] Calcraft P. J. . NAADP mobilizes calcium from acidic organelles through two-pore channels. Nature 459, 596–600, doi: 10.1038/nature08030 (2009). 19387438 PMC2761823

[b14] Ruas M. . Expression of Ca2+ -permeable two-pore channels rescues NAADP signalling in TPC-deficient cells. The EMBO journal, doi: 10.15252/embj.201490009 (2015). PMC4516428 25872774

[b15] Pitt S. J. . TPC2 is a novel NAADP-sensitive Ca2+ release channel, operating as a dual sensor of luminal pH and Ca2+. The Journal of biological chemistry 285, 35039–35046, doi: 10.1074/jbc.M110.156927 (2010). 20720007 PMC2966118

[b16] Brailoiu E. . An NAADP-gated two-pore channel targeted to the plasma membrane uncouples triggering from amplifying Ca2+ signals. The Journal of biological chemistry 285, 38511–38516, doi: 10.1074/jbc.M110.162073 (2010). 20880839 PMC2992283

[b17] Schieder M., Rotzer K., Bruggemann A., Biel M. & Wahl-Schott C. A. Characterization of two-pore channel 2 (TPCN2)-mediated Ca2+ currents in isolated lysosomes. The Journal of biological chemistry 285, 21219–21222, doi: 10.1074/jbc.C110.143123 (2010). 20495006 PMC2898409

[b18] Yamaguchi S. . Transient receptor potential mucolipin 1 (TRPML1) and two-pore channels are functionally independent organellar ion channels. The Journal of biological chemistry 286, 22934–22942, doi: 10.1074/jbc.M110.210930 (2011). 21540176 PMC3123061

[b19] Pereira G. J. . NAADP-sensitive two-pore channels are present and functional in gastric smooth muscle cells. Cell calcium 56, 51–58, doi: 10.1016/j.ceca.2014.04.005 (2014). 24882212

[b20] Macgregor A. . NAADP controls cross-talk between distinct Ca2+ stores in the heart. The Journal of biological chemistry 282, 15302–15311, doi: 10.1074/jbc.M611167200 (2007). 17387177

[b21] Zhang F. & Li P. L. Reconstitution and characterization of a nicotinic acid adenine dinucleotide phosphate (NAADP)-sensitive Ca2+ release channel from liver lysosomes of rats. The Journal of biological chemistry 282, 25259–25269, doi: 10.1074/jbc.M701614200 (2007). 17613490

[b22] Wolf I. M. . Frontrunners of T cell activation: Initial, localized Ca2+ signals mediated by NAADP and the type 1 ryanodine receptor. Sci Signal 8, ra102, doi: 10.1126/scisignal.aab0863 (2015). 26462735

[b23] Dammermann W. & Guse A. H. Functional ryanodine receptor expression is required for NAADP-mediated local Ca2+ signaling in T-lymphocytes. The Journal of biological chemistry 280, 21394–21399, doi: 10.1074/jbc.M413085200 (2005). 15774471

[b24] Nebel M. . Nicotinic acid adenine dinucleotide phosphate (NAADP)-mediated calcium signaling and arrhythmias in the heart evoked by beta-adrenergic stimulation. The Journal of biological chemistry 288, 16017–16030, doi: 10.1074/jbc.M112.441246 (2013). 23564460 PMC3668757

[b25] Naylor E. . Identification of a chemical probe for NAADP by virtual screening. Nature chemical biology 5, 220–226, doi: 10.1038/nchembio.150 (2009). 19234453 PMC2659327

[b26] Lam A. K. & Galione A. The endoplasmic reticulum and junctional membrane communication during calcium signaling. Biochimica et biophysica acta 1833, 2542–2559, doi: 10.1016/j.bbamcr.2013.06.004 (2013). 23770047

[b27] Helle S. C. . Organization and function of membrane contact sites. Biochimica et biophysica acta 1833, 2526–2541, doi: 10.1016/j.bbamcr.2013.01.028 (2013). 23380708

[b28] Berridge M. J. Calcium microdomains: organization and function. Cell calcium 40, 405–412, doi: 10.1016/j.ceca.2006.09.002 (2006). 17030366

[b29] Samanta K., Kar P., Mirams G. R. & Parekh A. B. Ca(2+) Channel Re-localization to Plasma-Membrane Microdomains Strengthens Activation of Ca(2+)-Dependent Nuclear Gene Expression. Cell reports 12, 203–216, doi: 10.1016/j.celrep.2015.06.018 (2015). 26146085 PMC4521080

[b30] Penny C. J., Kilpatrick B. S., Han J. M., Sneyd J. & Patel S. A computational model of lysosome-ER Ca2+ microdomains. Journal of cell science 127, 2934–2943, doi: 10.1242/jcs.149047 (2014). 24706947

[b31] Lopez-Sanjurjo C. I., Tovey S. C., Prole D. L. & Taylor C. W. Lysosomes shape Ins(1,4,5)P3-evoked Ca2+ signals by selectively sequestering Ca2+ released from the endoplasmic reticulum. J Cell Sci 126, 289–300, doi: 10.1242/jcs.116103 (2013). 23097044 PMC3603520

[b32] Fameli N., Ogunbayo O. A., van Breemen C. & Evans A. M. Cytoplasmic nanojunctions between lysosomes and sarcoplasmic reticulum are required for specific calcium signaling. F1000Research 3, 93, doi: 10.12688/f1000research.3720.1 (2014). 25126414 PMC4126599

[b33] Kinnear N. P. . Lysosomes co-localize with ryanodine receptor subtype 3 to form a trigger zone for calcium signalling by NAADP in rat pulmonary arterial smooth muscle. Cell calcium 44, 190–201, doi: 10.1016/j.ceca.2007.11.003 (2008). 18191199 PMC3982125

[b34] Kilpatrick B. S., Eden E. R., Schapira A. H., Futter C. E. & Patel S. Direct mobilisation of lysosomal Ca2+ triggers complex Ca2+ signals. Journal of cell science 126, 60–66, doi: 10.1242/jcs.118836 (2013). 23108667 PMC4208295

[b35] Davidson S. M. . Inhibition of NAADP signalling on reperfusion protects the heart by preventing lethal calcium oscillations via two-pore channel 1 and opening of the mitochondrial permeability transition pore. Cardiovascular research 108, 357–366, doi: 10.1093/cvr/cvv226 (2015). 26395965 PMC4648198

[b36] Neiss W. F. The electron density of light and dark lysosomes in the proximal convoluted tubule of the rat kidney. Histochemistry 77, 63–77 (1983). 6188724 10.1007/BF00496637

[b37] Schmucker D. L. & Sachs H. Quantifying dense bodies and lipofuscin during aging: a morphologist’s perspective. Archives of gerontology and geriatrics 34, 249–261 (2002). 14764327 10.1016/s0167-4943(01)00218-7

[b38] Penny C. J., Kilpatrick B. S., Eden E. R. & Patel S. Coupling acidic organelles with the ER through Ca(2)(+) microdomains at membrane contact sites. Cell calcium 58, 387–396, doi: 10.1016/j.ceca.2015.03.006 (2015). 25866010

[b39] Endo Y., Furuta A. & Nishino I. Danon disease: a phenotypic expression of LAMP-2 deficiency. Acta neuropathologica 129, 391–398, doi: 10.1007/s00401-015-1385-4 (2015). 25589223

[b40] Botcherby E. J. . Fast measurement of sarcomere length and cell orientation in Langendorff-perfused hearts using remote focusing microscopy. Circulation research 113, 863–870, doi: 10.1161/CIRCRESAHA.113.301704 (2013). 23899961

[b41] Rosen D. . Analogues of the nicotinic acid adenine dinucleotide phosphate (NAADP) antagonist Ned-19 indicate two binding sites on the NAADP receptor. J Biol Chem 284, 34930–34934, doi: 10.1074/jbc.M109.016519 (2009). 19826006 PMC2787355

[b42] Barcelo-Torns M. . NAADP mediates ATP-induced Ca2+ signals in astrocytes. FEBS letters 585, 2300–2306, doi: 10.1016/j.febslet.2011.05.062 (2011). 21664355

[b43] Lewis A. M. . beta-Adrenergic receptor signaling increases NAADP and cADPR levels in the heart. Biochemical and biophysical research communications 427, 326–329, doi: 10.1016/j.bbrc.2012.09.054 (2012). 22995315

[b44] Qi H., Li L. & Shuai J. Optimal microdomain crosstalk between endoplasmic reticulum and mitochondria for Ca2+ oscillations. Scientific reports 5, 7984, doi: 10.1038/srep07984 (2015). 25614067 PMC4303883

[b45] Berkefeld H. . BKCa-Cav channel complexes mediate rapid and localized Ca2+ -activated K + signaling. Science (New York, N.Y.) 314, 615–620, doi: 10.1126/science.1132915 (2006). 17068255

[b46] Davis L. C. . NAADP activates two-pore channels on T cell cytolytic granules to stimulate exocytosis and killing. Current biology: CB 22, 2331–2337, doi: 10.1016/j.cub.2012.10.035 (2012). 23177477 PMC3525857

[b47] Grimm C. . High susceptibility to fatty liver disease in two-pore channel 2-deficient mice. Nature communications 5, 4699, doi: 10.1038/ncomms5699 (2014). 25144390

[b48] Klionsky D. J., Eskelinen E. L. & Deretic V. Autophagosomes, phagosomes, autolysosomes, phagolysosomes, autophagolysosomes… wait, I’m confused. Autophagy 10, 549–551, doi: 10.4161/auto.28448 (2014). 24657946 PMC4091142

[b49] Bak J., Billington R. A., Timar G., Dutton A. C. & Genazzani A. A. NAADP receptors are present and functional in the heart. Current biology: CB 11, 987–990 (2001). 11448777 10.1016/s0960-9822(01)00269-x

[b50] Dammermann W. . NAADP-mediated Ca2+ signaling via type 1 ryanodine receptor in T cells revealed by a synthetic NAADP antagonist. Proceedings of the National Academy of Sciences of the United States of America 106, 10678–10683, doi: 10.1073/pnas.0809997106 (2009). 19541638 PMC2697110

[b51] Trabbic C. J., Zhang F., Walseth T. F. & Slama J. T. Nicotinic Acid Adenine Dinucleotide Phosphate Analogues Substituted on the Nicotinic Acid and Adenine Ribosides. Effects on ReceptorMediated Ca(2)(+) Release. Journal of medicinal chemistry 58, 3593–3610, doi: 10.1021/acs.jmedchem.5b00279 (2015). 25826221 PMC4480638

[b52] Cordiglieri C. . Nicotinic acid adenine dinucleotide phosphate-mediated calcium signalling in effector T cells regulates autoimmunity of the central nervous system. Brain: a journal of neurology 133, 1930–1943, doi: 10.1093/brain/awq135 (2010). 20519328 PMC2892943

[b53] Connolly M. J., Prieto-Lloret J., Becker S., Ward J. P. & Aaronson P. I. Hypoxic pulmonary vasoconstriction in the absence of pretone: essential role for intracellular Ca2+ release. The Journal of physiology 591, 4473–4498, doi: 10.1113/jphysiol.2013.253682 (2013). 23774281 PMC3784194

[b54] Nebel M. . Calcium Signalling Triggered by NAADP in T Cells Determines Cell Shape and Motility During Immune Synapse Formation. Messenger (Los Angeles, Calif.: Print) 4, 104–111, doi: 10.1166/msr.2015.1045 (2015). PMC5065091 27747143

[b55] Scriven D. R., Asghari P. & Moore E. D. Microarchitecture of the dyad. Cardiovascular research 98, 169–176, doi: 10.1093/cvr/cvt025 (2013). 23400762

[b56] Zhu M. X. . Calcium signaling via two-pore channels: local or global, that is the question. American journal of physiology. Cell physiology 298, C430–441, doi: 10.1152/ajpcell.00475.2009 (2010). 20018950 PMC2838574

[b57] Kinnear N. P., Boittin F. X., Thomas J. M., Galione A. & Evans A. M. Lysosome-sarcoplasmic reticulum junctions. A trigger zone for calcium signaling by nicotinic acid adenine dinucleotide phosphate and endothelin-1. The Journal of biological chemistry 279, 54319–54326, doi: 10.1074/jbc.M406132200 (2004). 15331591

[b58] Fernandez-Suarez M. & Ting A. Y. Fluorescent probes for super-resolution imaging in living cells. Nature reviews. Molecular cell biology 9, 929–943, doi: 10.1038/nrm2531 (2008). 19002208

[b59] Soubannier V. . A vesicular transport pathway shuttles cargo from mitochondria to lysosomes. Current biology: CB 22, 135–141, doi: 10.1016/j.cub.2011.11.057 (2012). 22226745

[b60] Skepper J. N. & Navaratnam V. Lipofuscin formation in the myocardium of juvenile golden hamsters: an ultrastructural study including staining for acid phosphatase. Journal of anatomy 150, 155–167 (1987). 3654330 PMC1261672

[b61] Zhang J. Teaching the basics of autophagy and mitophagy to redox biologists–mechanisms and experimental approaches. Redox biology 4, 242–259, doi: 10.1016/j.redox.2015.01.003 (2015). 25618581 PMC4803799

[b62] Lemasters J. J., Theruvath T. P., Zhong Z. & Nieminen A. L. Mitochondrial calcium and the permeability transition in cell death. Biochimica et biophysica acta 1787, 1395–1401, doi: 10.1016/j.bbabio.2009.06.009 (2009). 19576166 PMC2730424

[b63] Ong S. B., Samangouei P., Kalkhoran S. B. & Hausenloy D. J. The mitochondrial permeability transition pore and its role in myocardial ischemia reperfusion injury. Journal of molecular and cellular cardiology 78, 23–34, doi: 10.1016/j.yjmcc.2014.11.005 (2015). 25446182

[b64] Rizzuto R., Brini M., Murgia M. & Pozzan T. Microdomains with high Ca2+ close to IP3-sensitive channels that are sensed by neighboring mitochondria. Science (New York, N.Y.) 262, 744–747 (1993). 10.1126/science.8235595 8235595

[b65] Csordas G. . Structural and functional features and significance of the physical linkage between ER and mitochondria. The Journal of cell biology 174, 915–921, doi: 10.1083/jcb.200604016 (2006). 16982799 PMC2064383

[b66] Powell T., Terrar D. A. & Twist V. W. Electrical properties of individual cells isolated from adult rat ventricular myocardium. The Journal of physiology 302, 131–153 (1980). 6251204 10.1113/jphysiol.1980.sp013234 PMC1282839

[b67] Karnovsky M. J. A formaldehyde-glutaraldehyde fixative of high osmolality for use in electron microscopy. J Cell Biol 27, 137–138A (1965).

[b68] Spurr A. R. A low-viscosity epoxy resin embedding medium for electron microscopy. J Ultrastruct Res 26, 31–43 (1969). 4887011 10.1016/s0022-5320(69)90033-1

[b69] Kremer J. R., Mastronarde D. N. & McIntosh J. R. Computer visualization of three-dimensional image data using IMOD. Journal of structural biology 116, 71–76 (1996). 8742726 10.1006/jsbi.1996.0013

[b70] Rog-Zielinska E. A. . Electron tomography of rabbit cardiomyocyte three-dimensional ultrastructure. Progress in biophysics and molecular biology, doi: 10.1016/j.pbiomolbio.2016.05.005 (2016). PMC4959512 27210305

