# Supplementary material for: High resolution structural evidence suggests the Sarcoplasmic Reticulum forms microdomains with Acidic Stores (lysosomes) in the heart
Source: Sci Rep. 2017 Jan 17;7:40620. doi: 10.1038/srep40620 (PMC5240626; doi:10.1038/srep40620)
Supplement: Supplementary Information [file srep40620-s1.pdf]

High resolution structural evidence suggests the Sarcoplasmic Reticulum forms microdomains with Acidic Stores (lyososomes) in the heart.

Daniel Aston, Rebecca A. Capel, Kerrie L. Ford, Helen C. Christian, Gary R. Mirams, Eva A Rog-Zielinska, Peter Kohl, Antony Galione, Rebecca AB. Burton, Derek A. Terrar

Supplementary Information:

Supplementary Figures 1 – 5

Supplementary Video Figure Legend

Supplementary Methods

Supplementary Figure 1

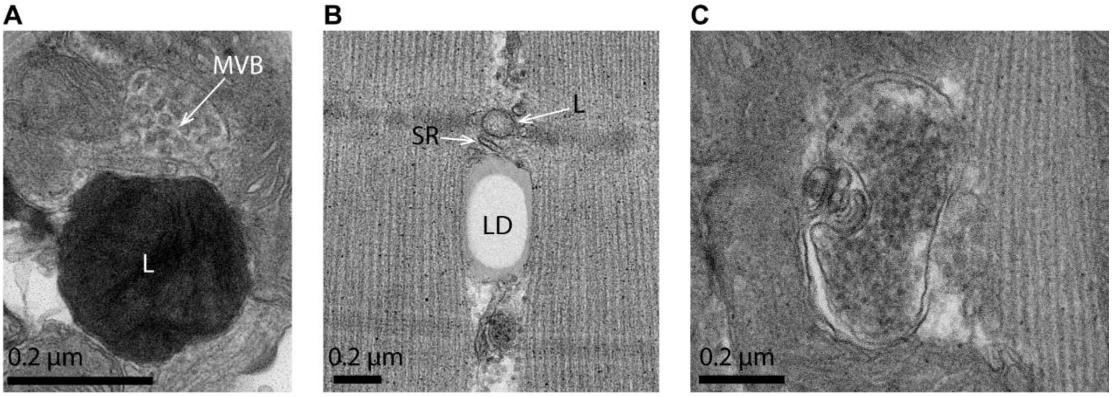

Supplementary Figure 1: Subcellular Organelles in Cardiac Ventricular Myocytes not included in our statistic counts. A) Example of a Multi-Vesicular Body (MVB). **B)** Example of a Lipid Droplet (LD). **C)** Example of an Autophagosome. Lysosomes (L), were included in the main analysis. Sarcoplasmic Reticulum (SR).

Supplementary Figure 2

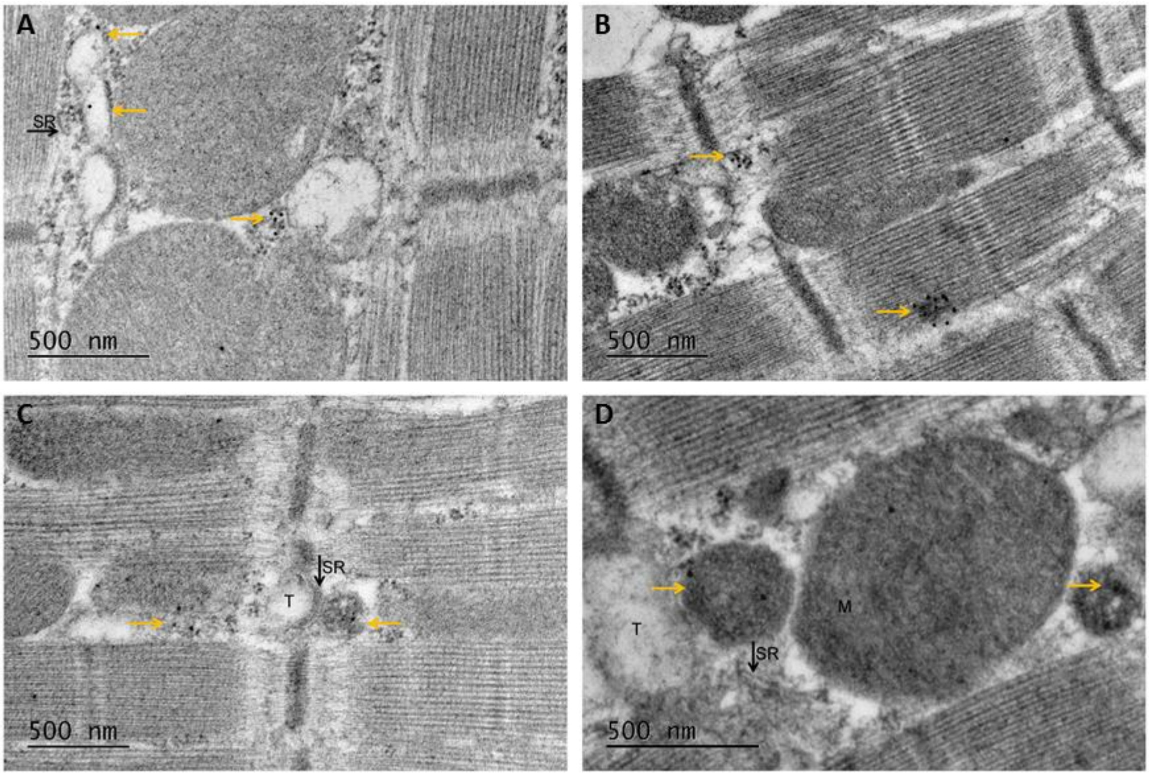

Supplementary Figure 2: Immuno-Gold Staining of LAMP-2 in Mouse Ventricular Tissue. A-D) LAMP-2 stained organelles (black dots are gold labelling, highlighted using yellow arrows) are found in close proximity to organelles likely to be Sarcoplasmic Reticulum (SR), mitochondria (M) and T-Tubules (T). This fixation method does not allow for accurate measurement of organelle separation.

Supplementary Figure 3

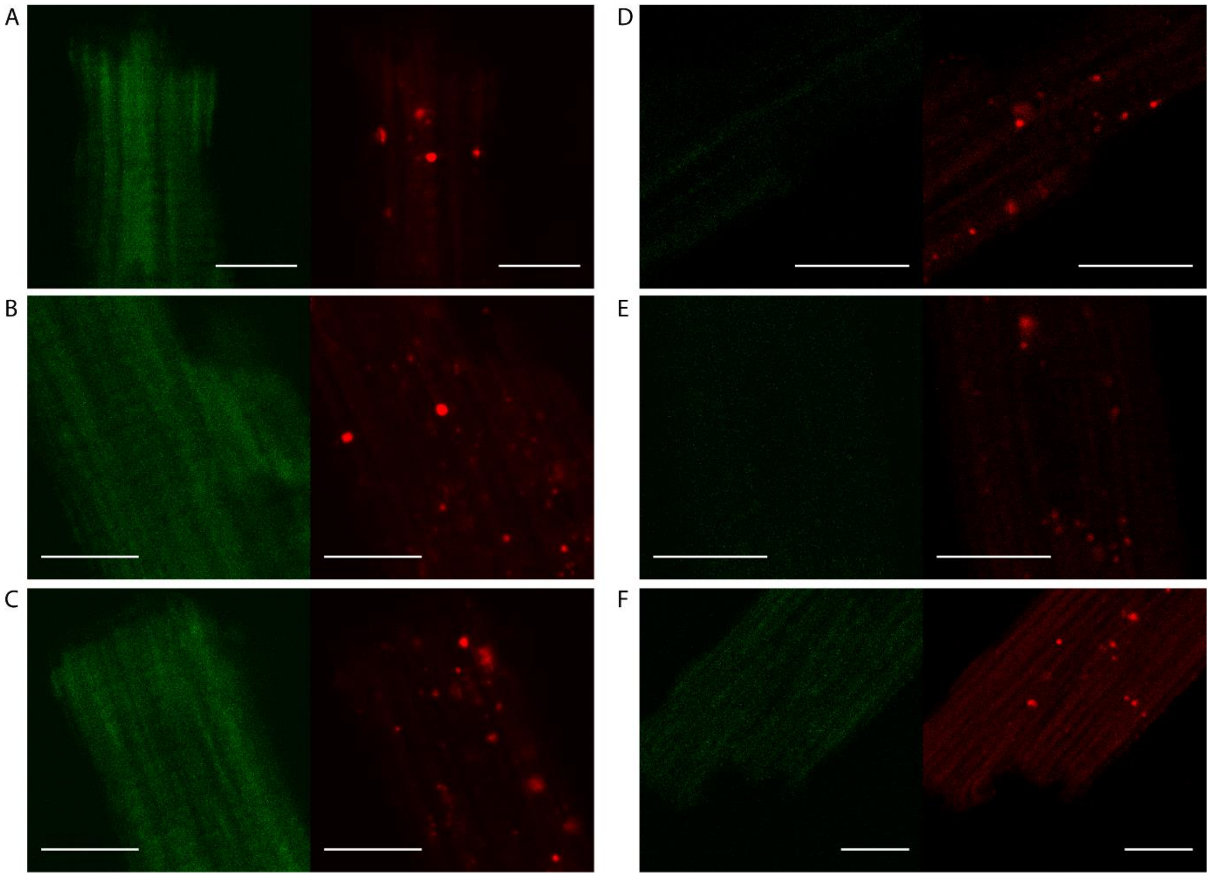

Supplementary Figure 3: Controls for Ned-19 and Lysotracker Co-localisation Studies in Live Ventricular Cardiomyocytes. All scale bars: 10 µm. **A to C)** Isolated rabbit ventricular myocytes loaded with Ned-19 for short durations not expected to produce staining (range 15-30 mins) and LysoTracker Red as per the methods section. Left, Ned-19 channel; Right, LysoTracker channel (raw images). Any autofluorescence in the Ned-19 channel is uniform and therefore cannot account for the punctate staining seen during co-localisation studies. **D to F)** Isolated rat ventricular myocytes loaded with LysoTracker only. Left, Ned-19 channel; Right, LysoTracker channel (raw images). LysoTracker signal peaks exhibit no bleedthrough to the Ned-19 channel and therefore cannot account for co-localisation of the signals seen in Figure 8.

Supplementary Figure 4

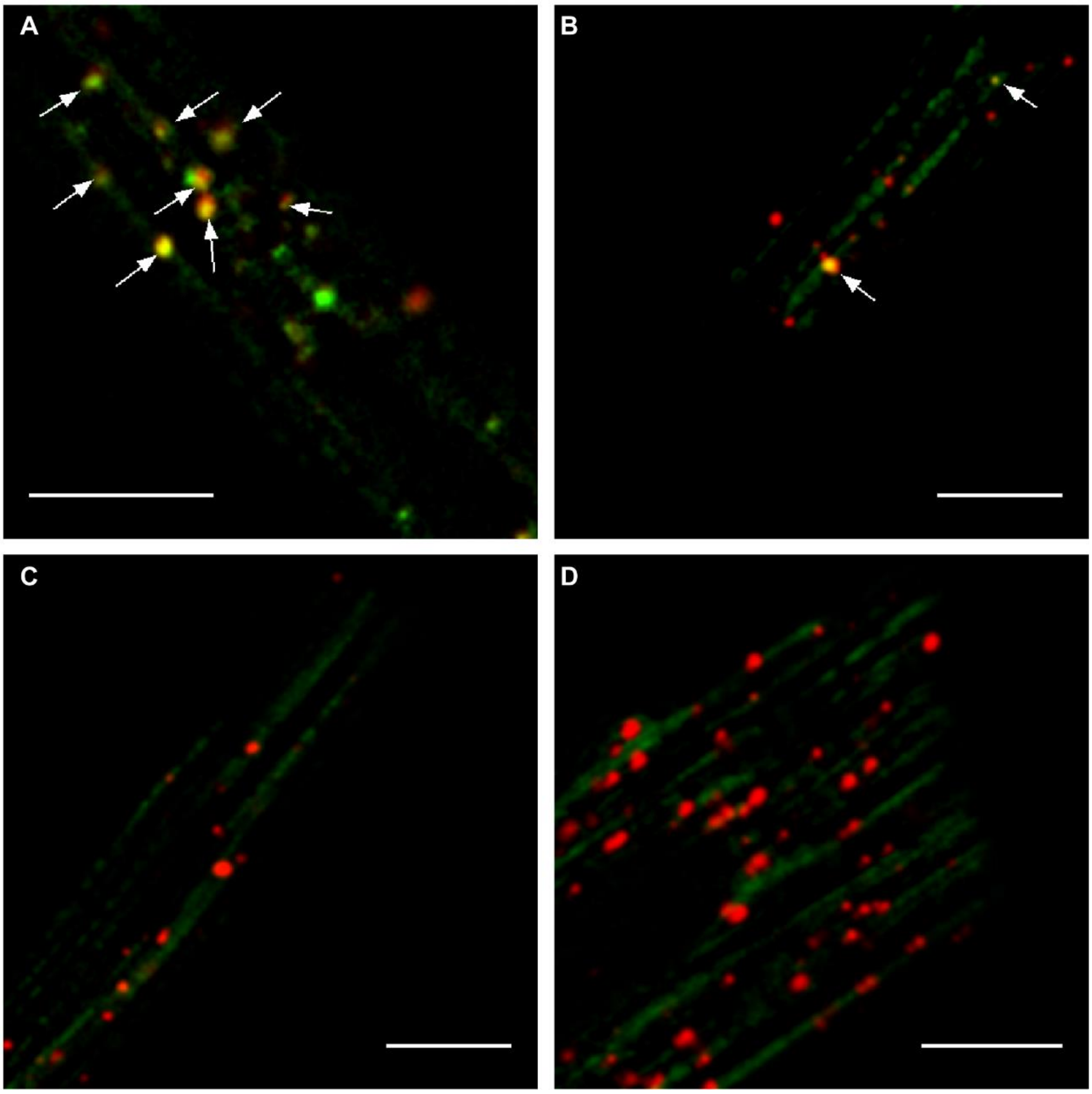

Supplementary Figure 4: Controls to Demonstrate Ned-19 Binding Specificity in Live Ventricular Cardiomyocytes. All images presented in this figure are merged, with Ned-19 channel signals in green and LysoTracker channel signals in red. Scale bars indicate 10 µm. **A)** Repeat of the Ned-19 and LysoTracker co-labelling experiment in the main paper. **B&C)** Ned-19 and LysoTracker loaded following pre-incubation with BZ194, a different published inhibitor of NAADP-mediated signalling. Of 19 cells imaged in this data set 3 cells exhibited the partial staining shown in B, whilst 16 exhibited no evidence of lysosomal Ned-19 as shown in C, demonstrating that BZ194 pre-incubation prevents Ned-19 localising to lysosomes. **D)** As a control for possible fluorescent interference of BZ194, cells were incubated with BZ194 and LysoTracker Red alone. These results show that BZ194 is not in itself fluorescent under the imaging conditions used for this study.

Ned-19 does not stain acidic stores after pre-incubation with another known inhibitor of NAADP signalling (BZ194), suggesting that Ned-19 is binding at the lysosomal membrane and not accumulating in acidic compartments as part of a degradative pathway. Scale bar: 10 µm.

Supplementary Figure 5

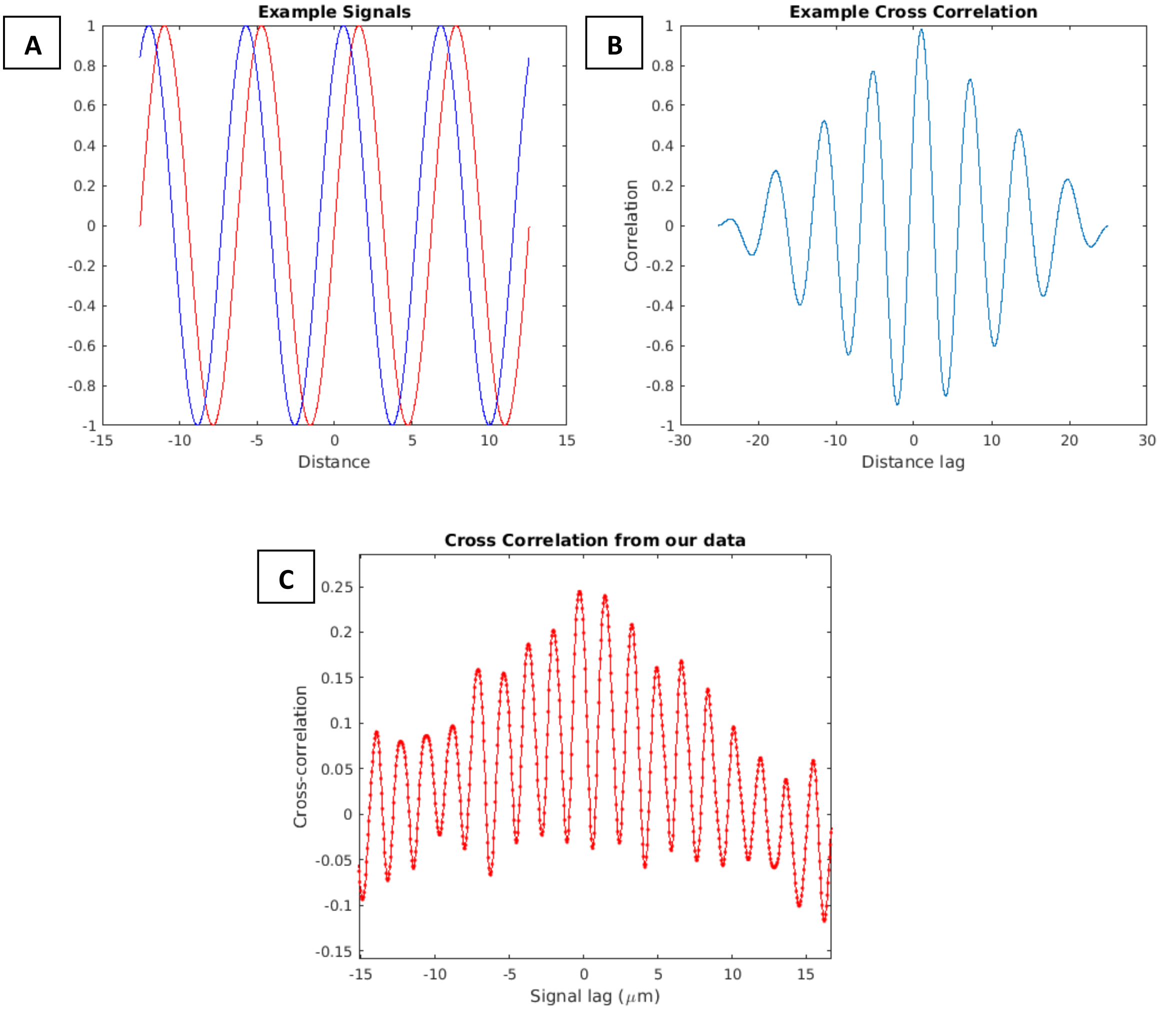

Supplementary Figure 5: Fourier/Pearson compatibility. Both RyR (or PLB) and LAMP show the same dominant wavelength – i.e. there are repeating peaks in the orientated images at the same spacing. There is not always a very good Pearson correlation because the images we have used are detailed enough to show that the signals are adjacent but not 'on top of each other'. In this Figure we show how the cross-correlation varies from positive (signal lag = 0) to more strongly positive when a phase shift (lag) in one signal is introduced, to negative when the signals are out of phase. This correlation varies from positive to negative, as expected, over the sarcomere length. So the Pearson correlation is maximised when a lag is introduced equivalent to the mean distance between RyR/PMB and LAMP, and there is no incongruity between the Pearson and Fourier analyses. **A)** Generated 'ideal' example signals with appropriate analysis of those signals in **B. C)** Phase-lag cross-correlation analysis of the data presented in this paper.

Supplementary Video 1

Dual axis electron tomography used to image, reconstruct and model lysosomes, T-tubules, mitochondria and sarcoplasmic reticulum (snap shots seen in Figure 5A&B). Plane-by-plane 3D electron tomography (00:00 to 00:30) is followed by zoomed video of nanojunction region. Reconstructions of a lysosome (red), SR (blue), a mitochondria (yellow) and T-tubule (green) super-imposed on original images (00:39 to 00:45). Multi-angle views of re-constructed organelles and spatial interactions (00:45 to end).

Supplementary Methods

Immuno-Gold Staining for Electron Microscopy

Samples were prepared from chemically fixed intact mouse ventricular tissue. Approximately 1 mm^3 pieces of tissue from ventricles were chemically fixed (paraformaldehyde 4%, glutaraldehyde 0.1%, phosphate buffer, pH 7.4), embedded in LR White resin and cut into ultrathin sections. For staining sections were washed in distilled water followed by PBS/chicken egg albumin (EA) twice for 5 minutes and blocked in EA for 30 minutes. Sections were incubated in LAMP-2 (rabbit-anti-LAMP-2, Thermofisher PA1-655, Lot: PA189864) primary antibody made up in PBS/EA (2 hours) and washed several times in PBS/EA followed by incubation with protein A gold (1 hour, British Biocell). Finally, sections were washed in PBS/EA and distilled water several times. Prior to viewing the section under an electron microscope (TEM, Joel 1200EX II), sections were post-stained with uranyl acetate and lead citrate.

Controls for Ned-19 Staining in Live Cells

The derivatized nicotinic acid BZ194 has been reported to antagonize NAADP-mediated Ca^{2+} signalling[1]. Cells were incubated with BZ194 (100 µM, 1 h) and Ned-19 (100 µM, 40 minutes) at room temperature with addition of 500 nM LysoTracker red for the final 2 min. Cells were allowed to adhere to a glass coverslip and superfused with Tyrode’s solution containing (in mM): 135 NaCl, 4.5 KCl, 11 glucose, 20 HEPES, 1 $MgCl_2$, 1.8 $CaCl_2$, pH 7.4 with NaOH. Ned-19 fluorescence was excited at 355 nm and detected at 415±30 nm, whilst LysoTracker red was excited at 514 nm and collected at 590-747 nm. Imaging was performed with a Leica SP5 inverted confocal microscope with a 63x oil-immersion objective.

References

1. Dammermann, W. *et al.* NAADP-mediated Ca^{2+} signaling via type 1 ryanodine receptor in T cells revealed by a synthetic NAADP antagonist. *Proc. Natl. Acad. Sci. U.S.A.* 106, 10678-10683 (2009)
